# Role of mitochondrial metabolic disorder and immune infiltration in diabetic cardiomyopathy: new insights from bioinformatics analysis

**DOI:** 10.1186/s12967-023-03928-8

**Published:** 2023-02-01

**Authors:** Cheng Peng, Yanxiu Zhang, Xueyan Lang, Yao Zhang

**Affiliations:** 1 Department of Cardiology, the Second Affiliated Hospital of Harbin Medical University, Harbin, 150001 China; 2 Key Laboratory of Myocardial Ischemia, Ministry of Education, Harbin Medical University, Harbin, 150001 China

**Keywords:** Diabetic cardiomyopathy, Mitochondria, Metabolism, Immune infiltration, Immunometabolism, Bioinformatics analysis

## Abstract

**Background:**

Diabetic cardiomyopathy (DCM) is one of the common cardiovascular complications of diabetes and a leading cause of death in diabetic patients. Mitochondrial metabolism and immune-inflammation are key for DCM pathogenesis, but their crosstalk in DCM remains an open issue. This study explored the separate roles of mitochondrial metabolism and immune microenvironment and their crosstalk in DCM with bioinformatics.

**Methods:**

DCM chip data (GSE4745, GSE5606, and GSE6880) were obtained from NCBI GEO, while mitochondrial gene data were downloaded from MitoCarta3.0 database. Differentially expressed genes (DEGs) were screened by GEO2R and processed for GSEA, GO and KEGG pathway analyses. Mitochondria-related DEGs (MitoDEGs) were obtained. A PPI network was constructed, and the hub MitoDEGs closely linked to DCM or heart failure were identified with CytoHubba, MCODE and CTD scores. Transcription factors and target miRNAs of the hub MitoDEGs were predicted with Cytoscape and miRWalk database, respectively, and a regulatory network was established. The immune infiltration pattern in DCM was analyzed with ImmuCellAI, while the relationship between MitoDEGs and immune infiltration abundance was investigated using Spearman method. A rat model of DCM was established to validate the expression of hub MitoDEGs and their relationship with cardiac function.

**Results:**

MitoDEGs in DCM were significantly enriched in pathways involved in mitochondrial metabolism, immunoregulation, and collagen synthesis. Nine hub MitoDEGs closely linked to DCM or heart failure were obtained. Immune analysis revealed significantly increased infiltration of B cells while decreased infiltration of DCs in immune microenvironment of DCM. Spearman analysis demonstrated that the hub MitoDEGs were positively associated with the infiltration of pro-inflammatory immune cells, but negatively associated with the infiltration of anti-inflammatory or regulatory immune cells. In the animal experiment, 4 hub MitoDEGs (Pdk4, Hmgcs2, Decr1, and Ivd) showed an expression trend consistent with bioinformatics analysis result. Additionally, the up-regulation of Pdk4, Hmgcs2, Decr1 and the down-regulation of Ivd were distinctly linked to reduced cardiac function.

**Conclusions:**

This study unraveled the interaction between mitochondrial metabolism and immune microenvironment in DCM, providing new insights into the research on potential pathogenesis of DCM and the exploration of novel targets for medical interventions.

**Supplementary Information:**

The online version contains supplementary material available at 10.1186/s12967-023-03928-8.

## Background

With the changing lifestyles, the incidence of diabetes mellitus (DM) shows a rapidly increasing trend. As estimated by the International Diabetes Federation, the number of DM patients will be increased to 0.5784 billion by 2030, resulting in a morbidity of up to 10.2% [1]. DM increases the risk of developing heart failure (HF) by 2–4 times, as compared to healthy people [2], and thus it tends to cause a highly poor prognosis. Diabetic cardiomyopathy (DCM) is one of the severe cardiovascular complications [3] of DM first reported by S Rubler in 1972 [4]. It is multi-factorial in pathophysiology and has not yet been fully explored.

Increasing studies have noted that mitochondrial events that lead to damage and dysfunction, including abnormal dynamics [5], mitophagy [6–8], calcium homeostasis imbalance [9–11], disturbed energy metabolism and oxidative stress [12, 13], play an essential role in DCM. In addition, excessive accumulation of lipid intermediary metabolites is considered as directly linked to the toxic injury and dysfunction of diabetic myocardium [14]. It has been established that the immune infiltration and activation of inflammatory processes in myocardial tissues are also critical pathogeneses of DCM. For example, both type 1 and 2 DM models had increased myocardial infiltration of monocytes and macrophages [15, 16]; chronic hyperglycemia induced elevation of Th1, Th2, and Th17 cytokines by activating T cells via the RAGE-dependent pathway [17]; additionally, a high-glucose environment could also activate mast cells and induce the release of pro-inflammatory mediators, resulting in exacerbation of the pathological remodeling in DCM [18].

Interestingly, accumulating evidence has revealed that there is a potential link between immunity and mitochondrial metabolism, and the metabolic state can affect the development of inflammation through changing the immune microenvironment [19]. Typical T cell activation is accompanied by the up-regulation of insulin receptors and glycolytic enzymes [20].High levels of insulin can impair the function of regulatory T cells (Tregs) and inhibit their suppressive function towards inflammatory response via regulating the AKT/mTOR signaling pathway [21]. Both mitochondrial metabolism and immune-inflammation are key pathogeneses of DCM, but their crosstalk in DCM have not yet been reported and require further exploration.

Bioinformatics allows for screening of molecules which show a difference between patients and healthy individuals from microarray data that vary at multiple levels. It is appreciated as an effective research method for exploring the potential molecular mechanism of disease. With this method, the current study analyzed how mitochondria-related genes promote the development of DCM and correlate to the immune infiltration based on associated microarray data from GEO database (GSE4745, GSE5606, and GSE6880). Additionally, the relationship between hub mitochondria-related genes and immune infiltrates in DCM was investigated to help better understand the underlying immunometabolism during disease development.

## Methods

### Microarray data retrieval

DCM datasets were obtained from the public repository NCBI GEO (http://www.ncbi.nlm.nih.gov/geo) [22] using "diabetic cardiomyopathy" and "diabetic heart" as the search queries. We screened them further based on information such as sequencing type (transcriptology), animal species (Rattus norvegicus), sample source (ventricle), and modeling time. Finally, GSE4745, GSE5606 and GSE6880 were obtained. The GSE4745 ([RG_U34A] Affymetrix Rat Genome U34 Array) is generated by the GPL85 platform that contains 24 left ventricular (LV) samples from rattus norvegicus. To better analyze the differential genes between DCM group and control (CON) group, 8 samples collected on day 42, including 4 DCM samples and 4 CON samples, were selected for analysis [23]. The GSE5606 ([Rat230_2] Affymetrix Rat Genome 230 2.0 Array) is generated by the GPL1355 platform and composed of 14 LV samples from DCM rats (n = 7) and CON rats (n = 7) [24]. The GSE6880 ([RAE230A] Affymetrix Rat Expression 230A Array) is generated by the GPL341 platform comprising 6 LV samples from DCM rats (n = 3) and CON rats (n = 3) [25].

### Acquisition of microarray data and identification of differentially expressed genes (DEGs)

Data of each microarray were accessed from GEO using R package "GEO query". DEGs from each microarray were obtained with R package "limma" as implemented by GEO2R online tool (https://www.ncbi.nlm.nih.gov/geo/geo2r/) [26], and all identified DEGs met p < 0.05 and |log2 (Fold-change)|≥ 1. Resulting DEGs were visualized by Volcano Plot using R package "ggplot2" [27] and Heatmap using R package "ComplexHeatmap" [28].

### Functional enrichment analysis

Gene Set Enrichment Analysis (GSEA) [29] was applied using R package "clusterProfiler" [30], with the "c2.cp.v7.2.symbols.gmt" (https://www.gsea-msigdb.org/gsea/msigdb/index.jsp) as the reference gene set, the number of permutations as 10,000, and the threshold of significance as 10. The results were visualized with R package "ggplot2" [27].

Gene Ontology (GO) and Kyoto Encyclopedia of Genes and Genomes (KEGG) pathway enrichment analyses were accomplished in DEGs with R package "clusterProfiler" [30], and the items with P < 0.05 in Benjamini–Hochberg test were regarded has having statistical significance. The results were visualized by Chordal and Circle graphs using R packages "ggplot2" [27] and "GOplot" [31].

### Identification of mitochondria-related DEGs (MitoDEGs)

The mitochondrial protein database, MitoCarta3.0 (http://www.broadinstitute.org/mitocarta) [32], was visited to obtain 1,140 mitochondria-localized genes. MitoDEGs were obtained via intersecting the DEGs from each microarray and the 1,140 mitochondria-localized genes using a Venn Diagram, and they were visualized as a Heatmap with R package "ggplot2" [27]. The overlapped MitoDEGs among the three microarrays were eventually obtained.

### Analysis of protein–protein interactions (PPI) and identification of Hub genes

The overlapped MitoDEGs were processed for PPI analysis with STRING database (https://string-db.org/) [33], and the resulting interactions were visualized as a network using Cytoscape 3.8.2 [34]. Hub MitoDEGs were screened out using the plug-ins CytoHubba and MCODE as implemented by the Cytoscape 3.8.2.

### Acquisition of genes potentially key to DCM and HF

The CTD database (http://ctdbase.org/) [35] assembles interaction data between chemicals, gene products, functional phenotypes, and diseases, affording great convenience to research into disease-associated environmental exposures and potential mechanisms of action of drugs. With the CTD data, the link between hub MitoDEGs and the risk of developing DCM or HF was analyzed.

### Prediction of a hub MitoDEGs-Transcription factors (TF) -miRNAs network

To explore the upstream regulators of hub MitoDEGs, TFs of hub MitoDEGs were predicted with the plug-in iRegulon of the Cytoscape 3.8.2 [36], and miRNAs of hub MitoDEGs were predicted using the miRWalk database (http://mirwalk.umm.uni-heidelberg.de) [37]. The hub MitoDEGs, resulting TFs and miRNAs were visualized as a network by the Cytoscape 3.8.2.

### Immune infiltration analysis

The gene matrices of GSE5606 and GSE6880 original datasets were combined using the Perl script and normalized after elimination of the batch effect and the heterogeneity induced by different platforms with R package "sva" [38]. The normalized gene expression matrix was used for further immune infiltration analysis. The GSE4745 dataset was excluded from the analysis, as the number of genes in GSE4745 was significantly less than that in the other two datasets, which might result in bias results.

The ImmuCellAI (http://bioinfo.life.hust.edu.cn/web/ImmuCellAI) estimates the infiltration abundance of 36 immune cell types based on RNA-Seq data or gene-expression profiles from microarray data [39].The normalized gene expression matrix was uploaded to the ImmuCellAI for analysis of immune infiltration, with Wilcoxon rank sum test used for between-group comparisons. Spearman correlation analysis was applied to explore the link between MitoDEGs/hub MitoDEGs and the immune cells.

### Construction of animal models with DCM

The animal procedure was performed in strict accordance with *The Guide for Care and Use of Laboratory Animals *[40] and with the approval from the Laboratory Animal Ethics Committee. Ten Sprague–Dawley male rats, weighing 200 ± 20 g, were housed in the laboratory animal center of our hospital. The Sprague–Dawley rat model of type 2 DM was generated using a high-fat diet combined with a low-dose STZ injection [41–43]. The rats were allowed to acclimate for 1 week with free access to diet and water ad libitum in an environment that provided a relative temperature of 24 ℃, a relative humidity of 50–60%, and a 12 h/12 h light/dark cycle. Following that, the rats were divided into the CON and DCM groups by random assignment. The total modeling time was 16 weeks. Rats in the CON group were fed normal diet, while rats in the DCM group were fed high-fat diet containing 60 kcal% fat, 20 kcal% protein, and 20 kcal% carbohydrate. After 4 weeks, streptozotocin (STZ) (40 mg/kg, Solarbio) was intraperitoneally injected in rats of the DCM group to induce T2DM, and citric acid buffer at the same dose was administrated in rats of the CON group. One week after injection, blood glucose was measured from the tail vein, and a random glucose level > 16.7 mmol/L was indicative of successful modeling. Tissue samples were obtained after another 12 weeks of feeding. Blood glucose and body weight were monitored during modeling, and echocardiography and measurement of tibia length were performed before sampling.

### Echocardiography

Rats were anesthesized with intraperitoneal injection of 30 mg/kg pentobarbital. Echocardiography was performed with the transducer of a high-resolution imaging system. LV parameters, including left ventricular ejection fraction (LVEF), fraction shortening (FS), left ventricular internal diameters at systole (LVIDs) and diastole (LVIDd), were measured from long- /short-axis images of the LV. Cardiac function was assessed by analysis of data of 3–5 cardiac cycles.

### RNA extraction and qRT-PCR

Total RNA was extracted from cardiac tissue using Trizol and reversely transcribed into cDNA using a reverse transcription kit (Roche). qRT-PCR was fulfilled with a SYBR Green (Roche). The primers used for amplification were shown in Table S1. Target gene expression relative to GAPDH gene was shown as 2^ΔΔCt^.

### Western blotting

Myocardial tissue was used to extract the samples, which were then boiled in a loading buffer for five minutes. 10% SDS-PAGE was used to separate the proteins. Primary antibodies were grown on the PVDF membranes overnight at 4 °C. The primary antibodies used in this study were Actin (Dilution 1:1000, M20011, Abmart), Pdk4 (Dilution 1:1000, YN5701, Immunoway), Hmgcs2 (Dilution 1:5000, ab137043, abcam), Decr1 (Dilution 1:1000, A13014, ABclonal), and Ivd (Dilution 1:2000, 10822-1-AP, ProteinTech). The membranes were incubated with the secondary antibody for 1 h at room temperature. Finally, the membranes were detected by the ECL system.

### Immunohistochemistry

Dewaxed, rehydrated, and antigen retrieval were performed on paraffin slices. Methanol was used to inactivate endogenous peroxidase for 15 min. The slices were then sealed after being treated with 5% BSA for 1 h, followed by an overnight incubation with primary antibodies. The primary antibodies used in this study were Pdk4 (Dilution 1:50, YN5701, Immunoway), Hmgcs2 (Dilution 1:200, ab137043, abcam) Decr1 (Dilution 1:50, A13014, ABclonal) and Ivd (Dilution 1:500, 10822-1-AP, ProteinTech). The paraffin slices were cultured with secondary antibodies the next day for one hour at 37 °C. Finally, the slides were stained with a DAB Detection Kit, and the counterstained with haematoxylin.

### Correlation between hub MitoDEGs and cardiac function

Correlation between hub MitoDEGs and LV parameters (EF%, FS%, and LVIDs) was analyzed using the Pearson algorithm, and the results were visualized with R package "ggplot2"[27].

### Statistical analysis

Data are presented as the mean ± standard deviation (SD) of four independent experiments, and were analyzed using GraphPad Prism 8.0 (GraphPad Inc, San Diego, USA). The Shapiro–Wilk test was used to check data normality. The student's t-test was used to evaluate the difference between the two groups when the data were in accordance with the normal distribution. P value < 0.05 was considered to be statistically significant.

## Results

### DEGs in DCM and functional enrichment analysis

**Fig. 1:**
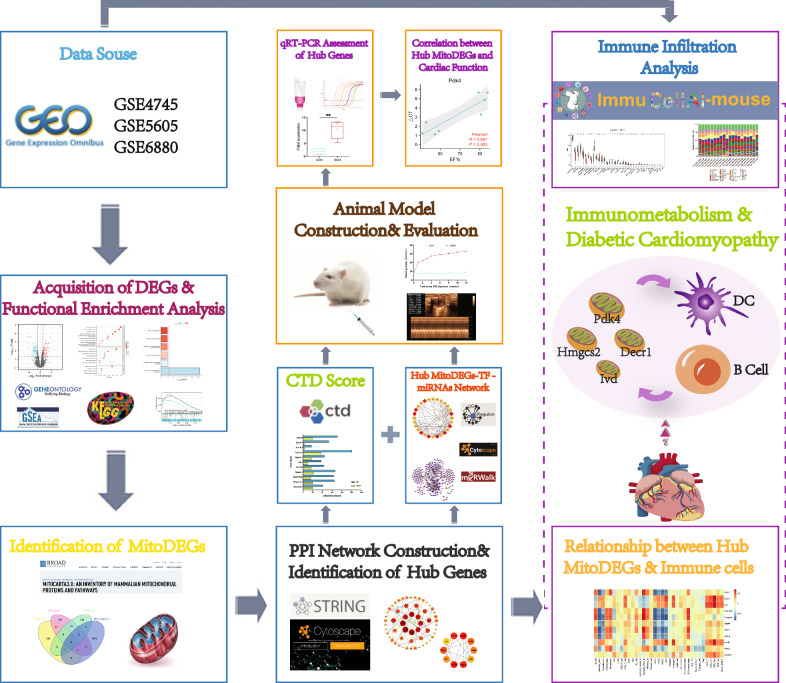
Flowchart of the multistep screening strategy on bioinformatics data

**Fig. 2:**
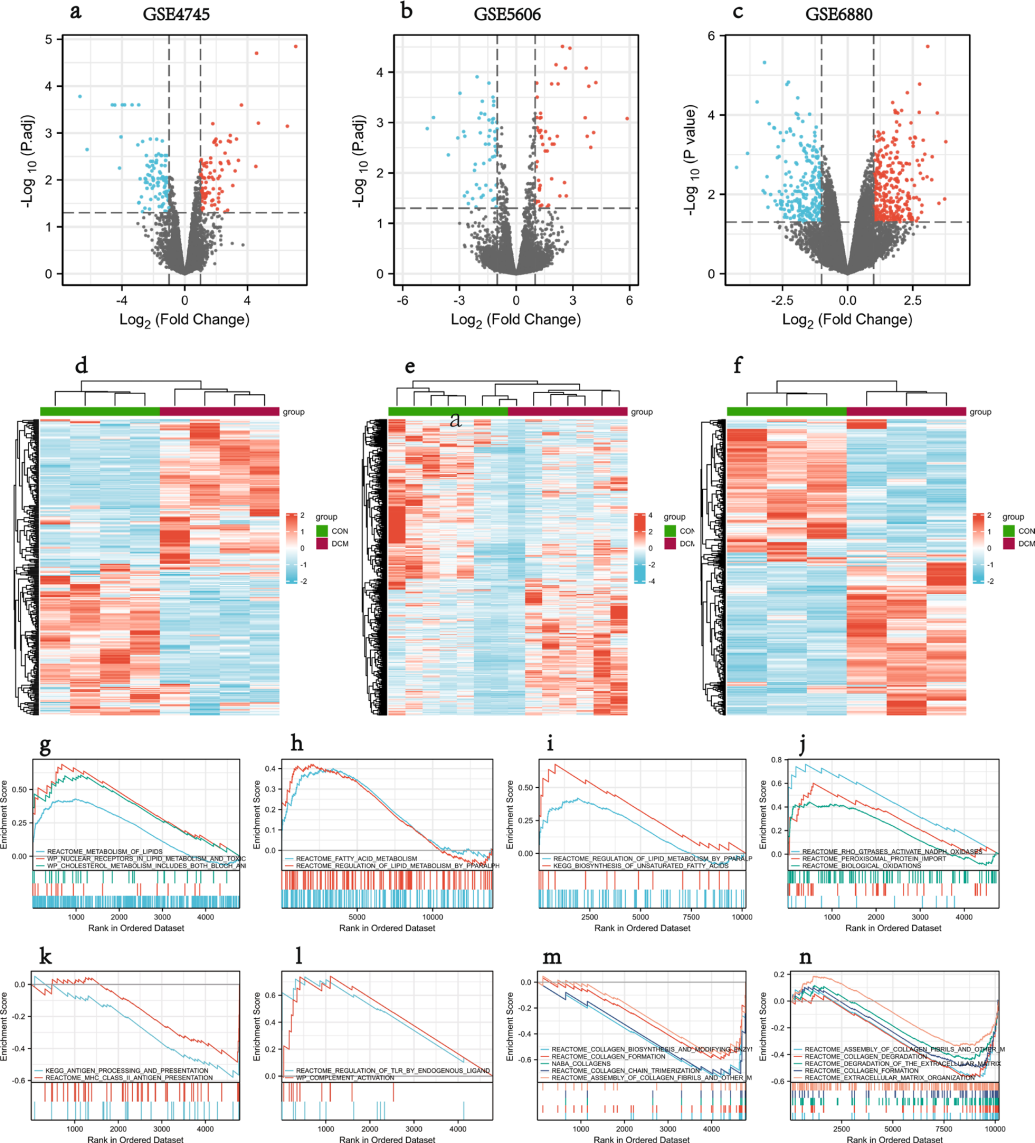
DEGs in DCM and results of GSEA analysis. **a**–**c** Volcano plot of DEGs in GSE4745, GSE5606, GSE6880; **d**–**f** Clustered heatmap of DEGs in GSE4745, GSE5606, GSE6880; **g**–**i** GSEA profiles depicting the 7 significant GSEA sets in lipid metabolism; **j** 3 significant GSEA sets in oxidative stress; **k**, **l** 4 significant GSEA sets in immunity; **m**, **n** 10 significant GSEA sets in collagen biosynthesis

Flowchart of overall data screening strategy was shown in Fig. 1. Three DCM-related GEO datasets, GSE4745, GSE5606, and GSE6880, were obtained for analysis. Differential analysis demonstrated 293 DEGs in the GSE4745 dataset, including 149 genes up-regulated and 144 genes down-regulated in DCM samples in comparison to normal samples; 544 DEGs in the GSE5606 dataset, including 269 up-regulated and 275 down-regulated genes; and 463 DEGs in the GSE6880 dataset, including 262 up-regulated and 201 down-regulated genes. The DEGs were visualized as Volcano Plots and Heatmaps (Fig. 2a–f).

GSEA showed that the DEGs from the three datasets were mainly involved in pathways related to lipid and fatty acid metabolism, and immunity, including Metabolism of lipids, Regulation of lipid metabolism by PPARα, Fatty acid metabolism, Biosynthesis of unsaturated Fatty acids, Antigen processing and presentation, MHC class II antigen presentation, Regulation of TLR by endogenous ligand, Complement activation (Fig. 2g–n). In addition, it also showed enrichment of pathways involved in collagen synthesis, collagen fibril assembly, and oxidative stress.

**Fig. 3:**
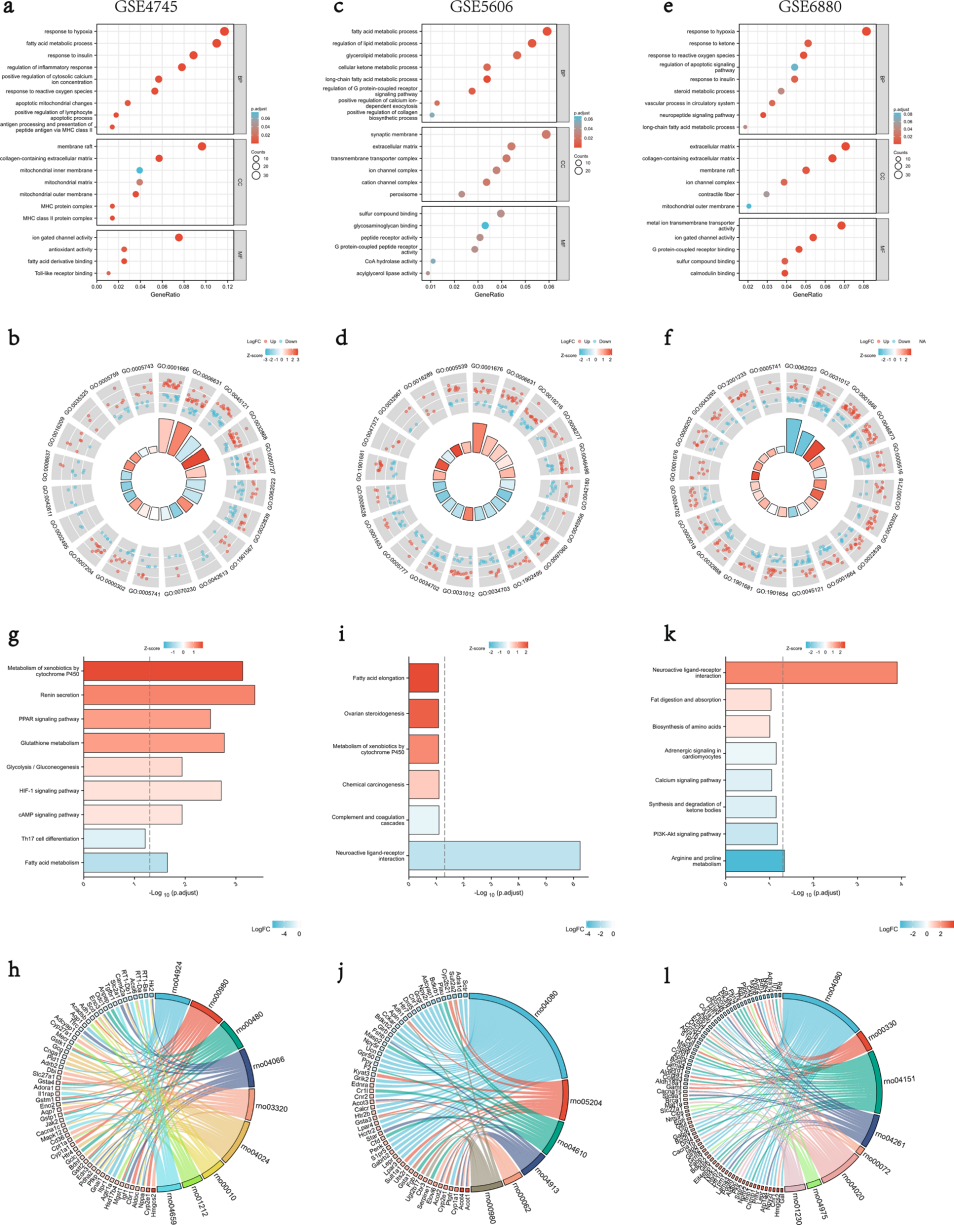
GO and KEGG enrichment analyses of DEGs from GSE4745, GSE5606 and GSE6880. **a**, **b** The enriched GO terms of DEGs in GSE4745; **c**, **d** The enriched GO terms of DEGs in GSE5606; **e**, **f** The enriched GO terms of DEGs in GSE6880; **g**, **h** KEGG pathway enrichment results in GSE4745; **i**, **j** KEGG pathway enrichment results in GSE5606; **k**, **l** KEGG pathway enrichment results in GSE6880. *BP* biological process, *CC* cellular component, *MF* molecular function

The DEGs were further processed for functional enrichment with GO and KEGG pathway analyses. The most enriched GO terms were classified to Biological Process (BP), Cellular Component (CC) and Molecular Function (MF), majoring including mitochondrial function and component, energy metabolism, inflammatory immunity, hypoxia and redox reaction, collagen synthesis, and insulin sensitivity, etc. (Fig. 3a–f). The most enriched KEGG pathways of the DEGs were dominated by pathways involved in mitochondrial metabolism and function, hypoxia and redox reaction, substance generation, and immunity, etc. (Fig. 3g–l).

### MitoDEGs in DCM

**Fig. 4:**
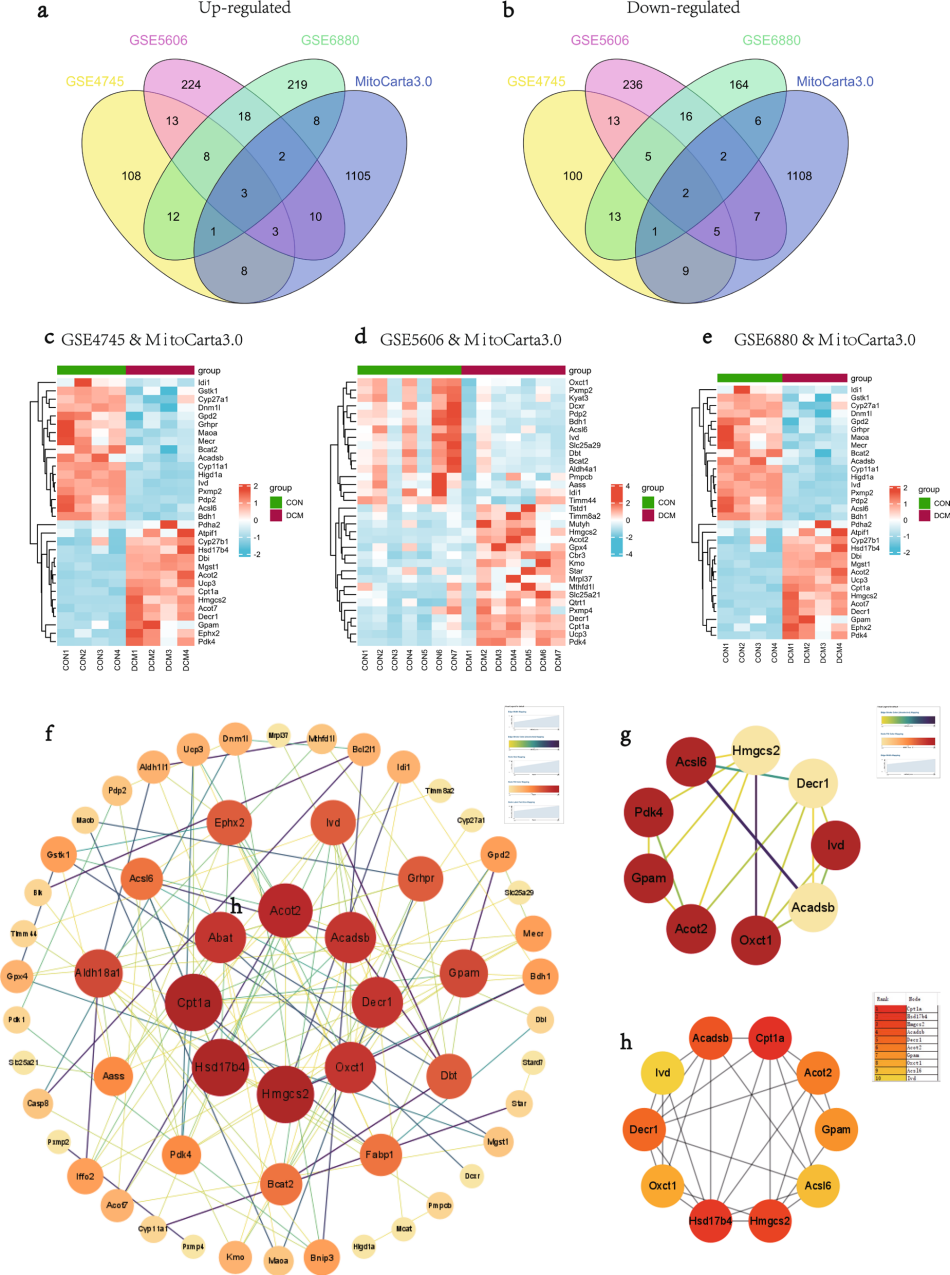
MitoDEGs in DCM; PPI network analysis and hub MitoDEGs identification. **a**, **b** Venn diagrams showed the number of upregulated **a** and downregulated **b** DEGs that overlap between GSE4745, GSE5606, GSE6880, MitoCarta3.0; **c** Clustered heatmap of DEGs both in GSE4745 and MitoCarta3.0; **d** Clustered heatmap of DEGs both in GSE5606 and MitoCarta3.0; **e** Clustered heatmap of DEGs both in GSE6880 and MitoCarta3.0; **f** PPI network of MitoDEGs; **g** A key cluster with 9 genes was further chosen as hub genes by MCODE; **h** Top 10 hub genes explored by CytoHubba

Mitochondria-related genes were retrieved from the MitoCarta3.0 database, and the genes overlapped with the DEGs from three datasets were selected as MitoDEGs. In total, there were 32 MitoDEGs (15 up-regulated and 17 down-regulated) in the GSE4745 datasets (Fig. 4c), 34 MitoDEGs (18 up-regulated and 16 down-regulated) in the GSE5606 datasets (Fig. 4d), and 25 MitoDEGs (14 up-regulated and 11 down-regulated) in the GSE6880 datasets (Fig. 4e). The MitoDEGs of each dataset were combined, resulting in 67 overlapped MitoDEGs, including 35 genes up-regulated and 32 genes down-regulated in DCM samples in comparison to normal samples.

### PPI network analysis and hub MitoDEGs identification

PPI of the 67 MitoDEGs was analyzed using the STRING database and visualized as a network with the Cytoscape (Fig. 4f). Significant modules (gene clusters) were identified using the plug-in MCODE as implemented by the Cytoscape with the following filter criteria: degree cut-off = 2; node score cut-off = 0.2; k-core = 2; and max depth = 100. A module that was composed of 9 nodes and 17 edges was identified as significant, and the genes involved in the module were Acsl6, Acadsb, Decr1, Ivd, Oxct1, Gpam, Pdk4, Hmgcs2, and Acot2 (Fig. 4g). With the MCC algorithm of plug-in CytoHubba, 10 candidate hub genes were identified from the PPI network, including Cpt1a, Hsd17b4, Hmgcs2, Acadsb, Decr1, Acot2, Gpam, Oxct1, Acsl6, and Ivd (Fig. 4h). Combining the results, 11 hub MitoDEGs, including Acadsb, Hmgcs2, Hsd17b4, Gpam, Acot2, Ivd, Decr1, Cpt1a, Acsl6, Oxct1, and Pdk4, were eventually obtained.

### Relationship between hub MitoDEGs and DCM/HF

**Fig. 5:**
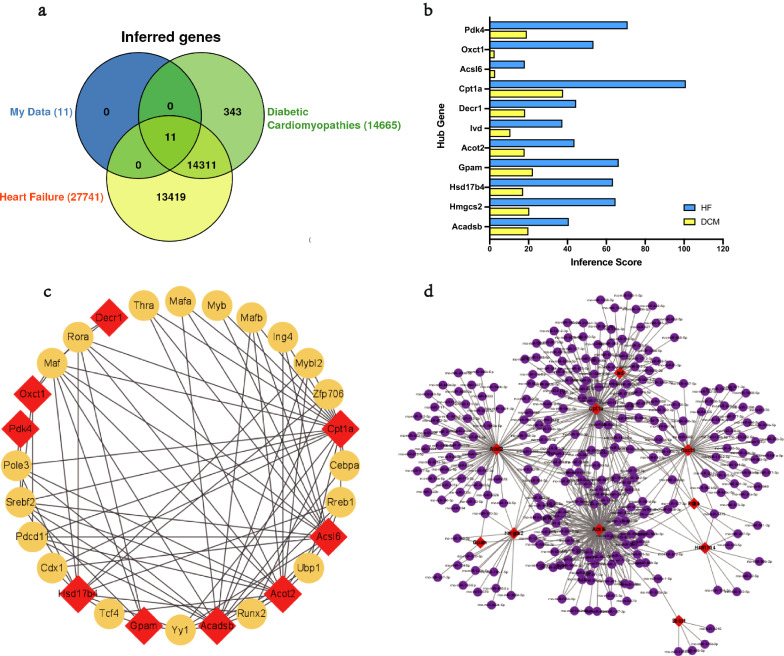
Relationship between hub MitoDEGs and DCM/HF; Hub MitoDEGs-TFs-miRNAs regulatory network. **a**, **b** Hub MitoDEGs related to DCM and HF diseases based on the CTD database; **c** TF–hub MitoDEGs regulatory network: the red squares represent hub MitoDEGs, and the yellow dots represent transcription factors; **d** miRNA–hub MitoDEGs regulatory network: the red squares represent hub MitoDEGs, and the purple dots represent miRNA

The CTD database was applied to predict the relationship between hub MitoDEGs and DCM/HF. As analyzed, Cpt1a, Gpam, Hmgcs2, and Acadsb had the highest association with DCM (Fig. 5a), while Cpt1a, Pdk4, Gpam, and Hmgcs2 showed the highest correlation with HF (Fig. 5b).

### Hub MitoDEGs-TFs-miRNAs regulatory network

The upstream regulation of the hub MitoDEGs was explored via predicting related TFs and miRNAs. TFs of hub MitoDEGs were predicted with plug-in iRegulon of the Cytoscape, and a hub MitoDEGs-TFs regulatory network comprising 19 TFs (Rora, Maf, Ing4, Srebf2, Mafb, Zfp706, Pole3, Rreb1, Mybl2, Myb, Tcf4, Mafa, Cebpa, Thra, Pdcd11, Yy1, Runx2, Cdx1, Ubp1) was constructed (Fig. 5c). miRNAs of hub MitoDEGs were predicted with the miRWalk 3.0, and a hub MitoDEGs-miRNAs regulatory network that involved 299 nodes and 569 edges was generated (Fig. 5d). There were three miRNAs, including miR-298-5p that had interactions with Ivd, Acsl6, Acot2, and Hmgcs2; miR-30c-1-3p that interacted with Oxct1, Ivd, Cpt1a, and Acsl6; and miR-344b-5p that interacted with Oxct1, Cpt1a, Acsl6, and Hmgcs2. However, further validation is required.

### Immune cell infiltration in DCM

**Fig. 6:**
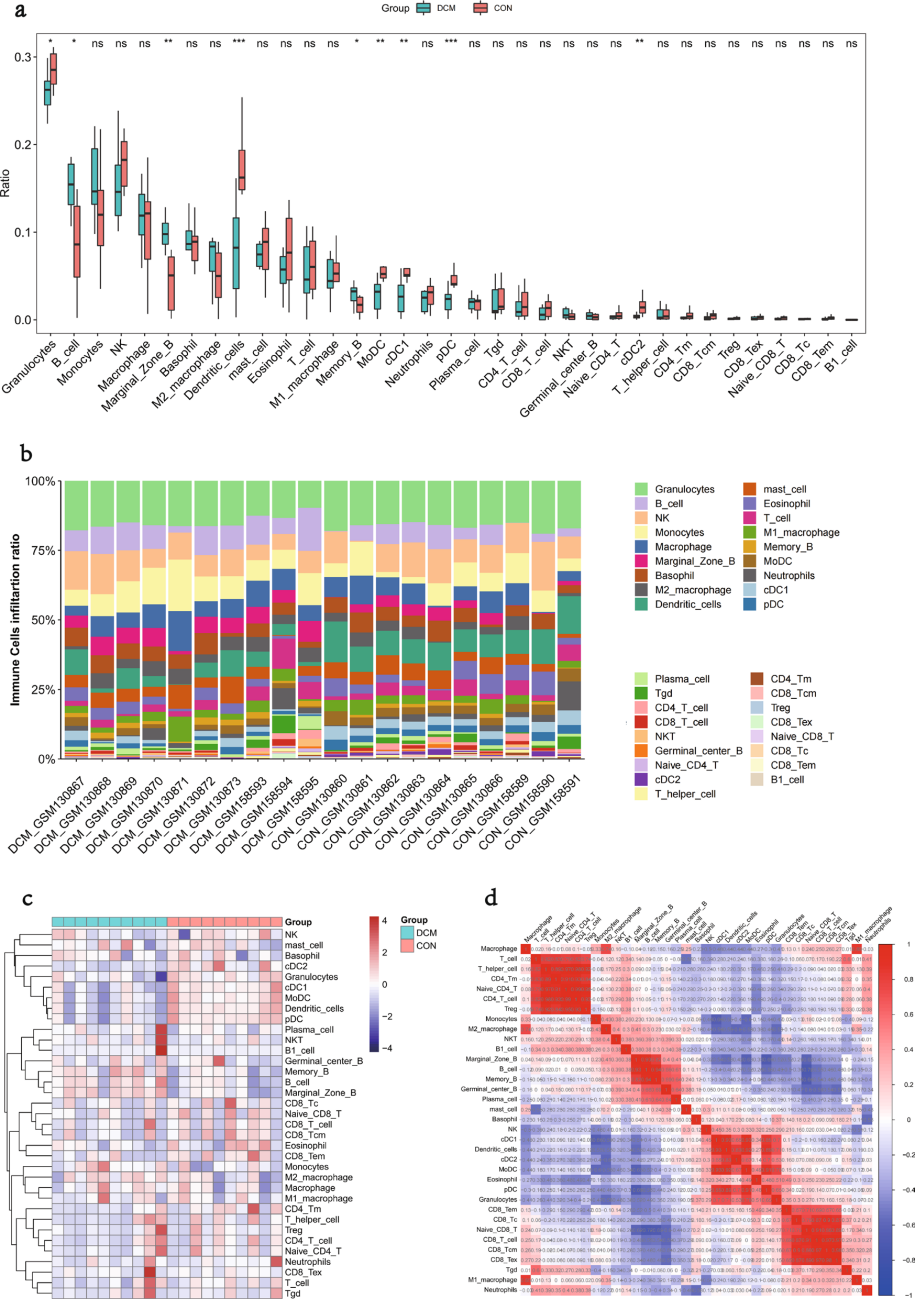
Infiltration of immune cell types compared between the DCM and CON. **a** The violin plot of the immune cell proportions; **b** Stacked bar chart of the immune cell; **c** Heatmap of the proportions of 36 immune cell types; **d** The correlation matrix of immune cell proportions

Infiltration of 36 immune cell types was analyzed using the ImmuCellAI algorithm and compared between the DCM and CON groups in the GSE5606 and GSE6880 datasets. Significant differences were demonstrated between the DCM and CON groups in the myocardial infiltration of 9 immune cell types (P < 0.05). Specifically, B cell, Marginal Zone B and Memory B were much more abundant in the DCM group, while Granulocytes, Dendritic cells, MoDC, cDC1, pDC, and cDC2 were more abundant in the CON group (Fig. 6a–c). Further analysis for the infiltrating immune cells in DCM showed multiple correlations between the cells (Fig. 6d). The degree of correlation was indicated by scores. The synergistic effect was observed as the strongest between CD4 T cell and Naive CD4 T (0.99), followed by CD4 T cell and T helper cell (0.98), CD8 Tcm and CD8 Tex (0.98), Naive CD4 T and T helper cell (0.97). In contrast, the competitive effect was found as the strongest between Naive CD8 T and B cell (-0.72), followed by pDC and Marginal Zone B (-0.69), Naive CD8 T and Memory B (-0.69).

### Relationship between MitoDEGs/hub MitoDEGs and immune cells

**Fig. 7:**
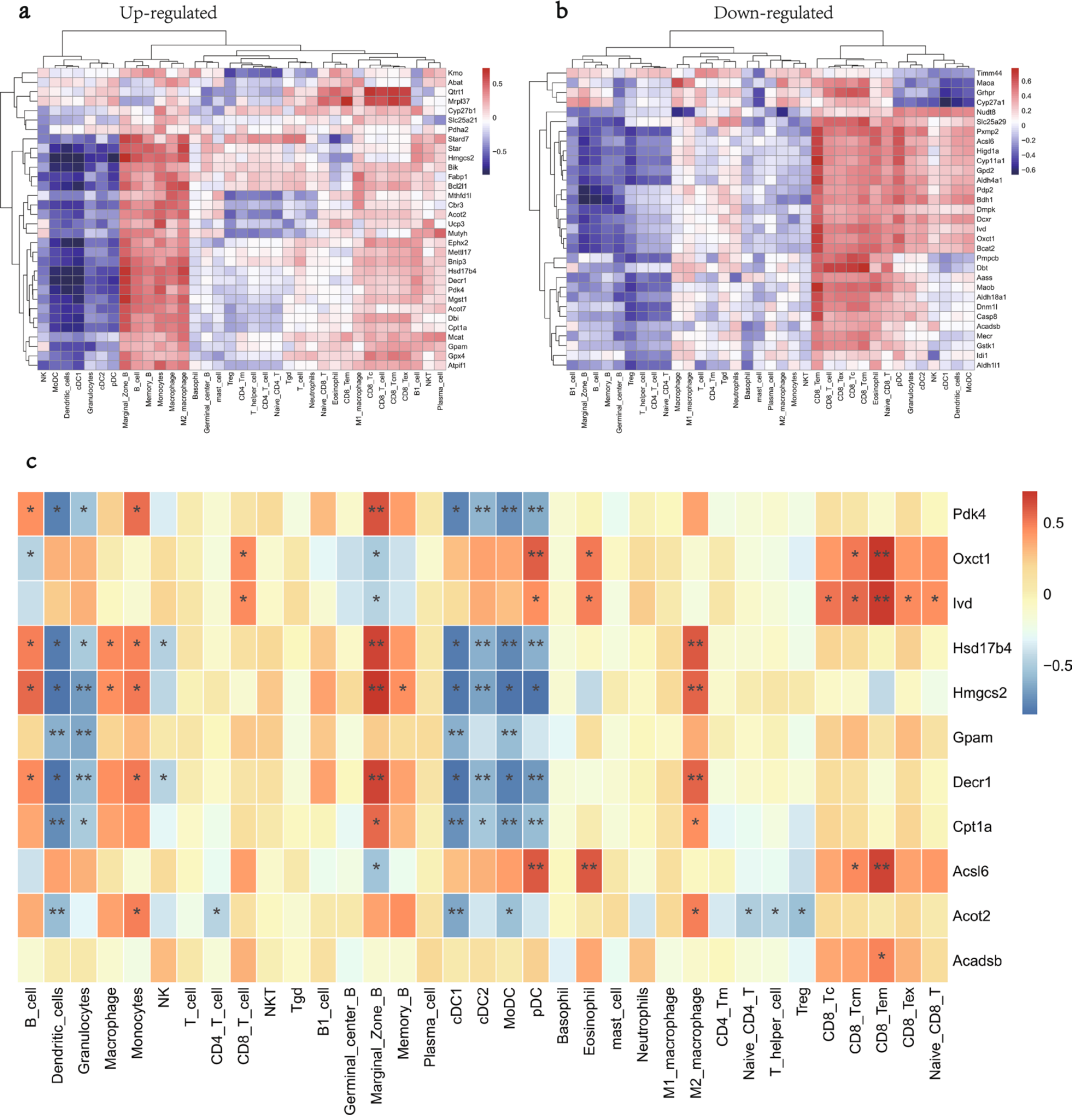
Relationship between MitoDEGs/hub MitoDEGs and immune cells. **a**, **b** The correlation between upregulated **a** and downregulated **b** DEGs and immune cells; **c** The correlation between hub MitoDEGs and immune cells

Spearman method was applied to explore the potential associations between MitoDEGs/hub MitoDEGs and immune cells. The positive/negative associations between MitoDEGs (35 up-regulated and 32 down-regulated) and immune cells were demonstrated in Fig. 7a, b. Of the 11 hub MitoDEGs, Pdk4 was positively associated with Marginal Zone B but negatively associated with cDC2, MoDC, and pDC; Oxct1 was positively associated with pDC and CD8 Tem; Ivd was positively associated with CD8 Tem; Hsd17b4 was positively associated with Marginal Zone B and M2 macrophage but negatively associated with cDC2, MoDC, and pDC; Hmgcs2 was positively associated with Marginal Zone B, M2 macrophage but negatively associated with Granulocytes and cDC2; Gpam was negatively associated with Dendritic cells, Granulocytes, cDC1, and MoDC; Decr1 was positively associated with Marginal Zone B and M2 macrophage while negatively associated with Granulocytes, cDC2, and pDC; Cpt1a was negatively associated with Dendritic cells, cDC1, MoDC, and pDC; Acsl6 was positively associated with pDC, Eosinophil, and CD8 Tem; Acot2 was negatively associated with Dendritic cells and cDC1 (Fig. 7c).

### General biological and echocardiography features of DCM rats

**Fig. 8:**
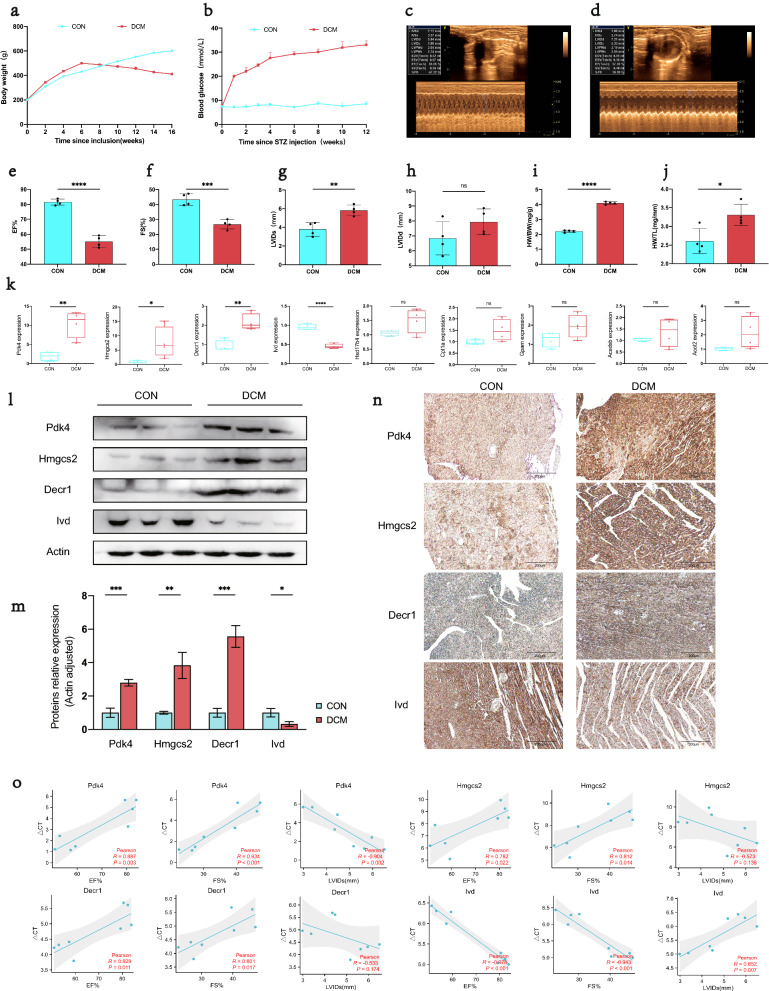
Confirmation of hub MitoDEGs expression and association with cardiac function in DCM rats. **a**–**j** General biological and echocardiography features of DCM rats; **k** Hub MitoDEGs mRNA expression of CON and DCM rats; **l**, **m** Protein levels of Pdk4, Hmgcs2, Decr1 and Ivd by western blotting, and quantitative analysis in cardiac tissues; **n** Immunostaining of Pdk4, Hmgcs2, Decr1 and Ivd protein expression. **o** Correlations between Pdk4, Hmgcs2, Decr1, Ivd mRNA levels and cardiac functional parameters in CON and DCM rats, including: EF%, FS%, LVIDs(mm). Mean ± SD, n = 4 rats per group. *p < 0.05, **p < 0.01, ***p < 0.001 and ****p < 0.0001 vs. control group. *ns* no significance

During modeling, the body weight of high-fat diet fed rats of the DCM group was significantly higher than that of the CON group, and it tended to decrease from 2 weeks after STZ injection and became remarkably lower than that of the CON group before tissue sampling (Fig. 8a). After 1 week of STZ induction, the blood glucose of the DCM group began to increase, and the level was consistently higher than that of the CON group throughout the entire modelling process (Fig. 8b). Echocardiography showed that as compared to the CON group, the DCM group witnessed significantly lower EF% and FS% (P < 0.05) but remarkably higher LVIDs (P < 0.05). Besides, the LVIDd was marginally varied between the two groups (Fig. 8c–h). Moreover, notable increases in the heart weight normalized to body weight (HW/BW) and heart weight normalized to tibia length (HW/TL) were found in the DCM group as compared to the CON group (P < 0.05, Fig. 8i, j).

### Experimental validations of hub MitoDEGs expression in DCM rats

Ventricular expression of 9 hub MitoDEGs (Acadsb, Acot2, Cpt1a, Decr1, Gpam, Hmgcs2, Hsd17b4, Ivd, and Pdk4) was validated in rats with qRT-PCR. As compared to the CON group, Pdk4, Hmgcs2 and Decr1 had significantly increased expression in the DCM group (P < 0.05), while Ivd reversely exhibited remarkably decreased expression in the DCM group (P < 0.05) (Fig. 8k). After that, we further validated the protein expressions of Pdk4, Hmgcs2, Decr1 and Ivd between the DCM and CON groups by western blotting and immunohistochemistry. The results showed that the protein expression levels of Pdk4, Hmgcs2, Decr1 and Ivd were consistent with those of mRNA (P < 0.05) (Fig. 8l–n).

### Relationship between hub MitoDEGs and cardiac function

The four hub MitoDEGs (Pdk4, Hmgcs2, Decr1, and Ivd) with distinct differential expression between the DCM and CON groups were further analyzed for their associations with EF%, FS% and LVIDs. The number of PCR cycles of Pdk4 had highly significant positive correlations with EF% (R = -0.904; P = 0.002) and FS% (R = 0.934; P < 0.001), but had a highly significant negative correlation with LVIDs (R = 0.852; P = 0.007); the number of PCR cycles of Hmgcs2 exhibited highly significant positive correlations with EF% (R = 0.782; P = 0.022) and FS% (R = 0.812; P = 0.014); the number of PCR cycles of Decr1 showed highly significant positive correlations with EF% (R = 0.829; P = 0.011) and FS% (R = 0.801; P = 0.017); the number of PCR cycles of lvd showed highly significant negative correlations with EF% (R = -0.978; P < 0.001) and FS% (R = -0.943; P < 0.001), but had a highly significant positive correlation with LVIDs (R = 0.852; P = 0.007) (Fig. 8o). Collectively, the up-regulated expression of Pdk4, Hmgcs2, and Decr1 and the down-regulated expression of Ivd in myocardial tissues of DCM were highly linked to the reduction in cardiac function.

## Discussion

The number of DM patients has grown worldwide at an alarming rate. DM commonly occurs with target organ damage that leads to a poor prognosis, and it is tightly linked to the initiation and development of HF [44]. It has been proven that the risk of developing HF in DM patients is associated with the presence of DCM [45]. However, it remains elusive about the pathogenesis of DCM, and there is a paucity of effective therapeutic strategies. In this context, strengthening our understanding on DCM pathogenesis and looking for potential therapeutic targets are in urgent need. With multiple bioinformatics methods, the present study firstly obtained DEGs from the three DCM-related microarray datasets from GEO and found that the DEGs were enriched in pathways associated with mitochondrial metabolism, immune-inflammation, and collagen synthesis. Mitochondrial dysfunction and metabolic abnormality have been proven to play a role in cardiac hypertrophy and myocardial fibrosis [46]. In addition, various activities of immune cells, such as transition from macrophages to fibroblast-like cells [47], B-cell infiltration [48], and transition between T lymphocyte subsets (Th17 to Treg) [49], are also critical for pathogenesis of myocardial fibrosis. Based on the findings, our study aimed at analyzing the regulatory roles of mitochondrial metabolism and immune dysregulation in the occurrence and development of DCM and exploring related targets. The findings of the study may help us better understand the mitochondrial metabolism, immunity, and their crosstalk in DCM.

Presently, mitochondria-related genes in DCM have not yet been reported by bioinformatics studies. For the first time, our study applied the MitoCarta 3.0, an authoritative database of mitochondrial proteome, to obtain mitochondria-related genes, and then identified 9 hub MitoDEGs with had a strong correlation with DCM or HF. To validate our findings, DCM rats were modeled. Expression analysis revealed four genes, including Pdk4, Hmgcs2, Decr1, and Ivd, which showed a consistent expression trend as that detected by prior bioinformatics analysis. Additionally, we found that the up-regulation of Pdk4, Hmgcs2, Decr1 and the down-regulation of Ivd were significantly associated with the reduction in cardiac function.

Mitochondrial metabolic disorder is one of the important pathogeneses of DCM [44], while Pdk4, Hmgcs2, Decr1, and Ivd are enzymes essential for mitochondrial metabolism. In DCM, the most significant metabolic disorders in myocardial tissues are decreased glucose utilization and increased fatty acid oxidation, which can lead to cardiac lipotoxicity, myocardial fibrosis, and effects on cardiac function. Pdk4 (Pyruvate dehydrogenase kinase 4) is localized to the mitochondrial matrix and participates in fatty acid oxidation as a key enzyme [50]. Studies found that Pdk4 showed increased expression in myocardial tissues of DM mice [51], and it could be used as a therapeutic target for DM [52, 53] due to its role as a key target genes of the PPARα signaling pathway [54, 55]. In addition, specific expression of Pdk4 could induce insulin resistance, reduction in myocardial glucose oxidation and increase in fatty acid oxidation [56, 57]. To the contrary, suppression of Pdk4 activity could lead to reduced mitochondria-associated ER membranes (MAM) formation and improve insulin signal transduction through preventing the MAM-induced mitochondrial Ca2 + accumulation [58]. Other than the role in mediating metabolic reprogramming, Pdk4 also has implications for cell respiration by playing a role in regulation of mitochondrial dynamics [59]. Hmgcs2 (3-hydroxy-3-methylglutaryl-CoA synthase 2) is also distributed to the mitochondrial matrix and acts as a rate-limiting enzyme in ketogenesis [60]. George A.Cook et al. [61] found that Hmgcs2 was increasingly expressed in DCM rats, consistent with the present study. Another study noted significantly increased expression of Hmgcs2 enzyme in the right ventricle in cases of arrhythmogenic cardiomyopathy, suggesting enhanced ketoacid metabolism, and it also reported concurrent elevation of plasm β-hydroxybutyrate. The results indicated that up-regulation of Hmgcs2 enzyme was predictive of occurrence of major adverse cardiovascular events and disease progression [62]. However, there was a study which demonstrated reduced cardiac content of Hmgcs2 in non-diabetic patients with end-stage HF [63]. We speculated that the discrepancy might be due to the difference in cardiac metabolic substrates between diabetic and non-diabetic cases [64]. Decr1 (2,4-dienoyl-CoA reductase 1) is a mitochondrial enzyme involved in degradation of poly-unsaturated fatty acids [65]. Most of the existing studies concentrated on its role in lipid metabolism in tumor cells [65–67], while only a few was performed in non-diabetic HF [68, 69]. Therefore, further research is in demand to explore the role of Decr1 in DCM. Ivd (Isovaleryl-CoA dehydrogenase) is another mitochondrial enzyme with implications for metabolism of the branched chain amino acids leucine [70]. Previous research revealed that leucine-enriched diet was conducive to improving the cardiac injury and dysfunction caused by cancer cachexia [71] and anti-tumor drugs [72]. Furthermore, circulating levels of branched chain amino acids were proven as independently associated with the incidence of HF in diabetic patients [73].

The metabolic status and immune processes are interconnected [74]. Immune dysregulation is common in DCM and plays a role in disease progression. In the present study, we used the ImmuCellAI algorithm to analyze immune cell infiltration and found higher enrichment of multiple dendritic cells (Dendritic cells, MoDC, cDC1, pDC, and cDC2) in the CON group than the DCM group. Dendritic cells are specialized antigen-presenting cells that serve as important mediators of immune responses [75], and the number was reduced in both type 1 and 2 DM patients [76, 77]. It was reported that dendritic cells were protective immunomodulators playing a role during the healing from myocardial infarction. In addition, dendritic cells tended to accumulate in infarct border zone after myocardial infarction and simultaneously mediated the regulation of homeostasis by monocytes and macrophages [78]. In human infarcted myocardial tissues, the reduced number of dendritic cells was reported as associated with the recruitment of pro-inflammatory monocytes, increase in macrophages, impairment of reparative fibrosis, and the cardiac rupture after myocardial infarction [79]. In all, dendritic cells protect the heart via regulating the recruitment of various types of immune cells. The current study also found that B cell, Marginal Zone B, and Memory B were highly abundant in the DCM group. B cells maintain the bridge between innate and adaptive immunity through their antigen-specific responses, and they are also conducive to sustaining the chronic inflammation in DCM [80]. Animal experiments revealed that B cells regulated the composition of the cardiac leukocyte pool, and B cell-deficient mice had a smaller fibrotic area while a higher LVEF [81]. Another study found that B cell depletion was accompanied by significant reductions in TNF-α, IL-1β, IL-18, and apoptosis in myocardial cells, and further introduction of B cells worsened inflammatory response and cardiac function [82]. Collectively, B cells are critical for the pro-inflammatory environment of the failing heart tissue and myocardial injury. There were also some studies showing that increase in neutrophil-to-lymphocyte ratio was associated with the incidence of subclinical DCM [83]; impaired Th/Treg balance and increased ventricular infiltration of T cells exacerbated the cardiac hypertrophy and fibrosis in T2DM [84, 85]; M1 macrophages potentiated DCM progression via secreting inflammatory factors to induce insulin resistance [86].

Mitochondrial metabolism can have a huge impact on the fate and function of immune cells. Correlation analysis of the study indicated that Pdk4, Hmgcs2, and Decr1 were positively associated Marginal Zone B while negatively associated with dendritic cells. In addition, Ivd was positively associated with CD8 Tem. This is consistent with our findings that dendritic cells had a lower enrichment in the DCM group than the CON group and had significant enrichment in B cells. The findings of the study deepen our understanding about the link between mitochondrial metabolism and immune cells in DCM.

In this study, the interaction between mitochondrial metabolism and the immune microenvironment was found for the first time through bioinformatics analysis of DCM. Screening and verification of Pdk4, Hmgcs2, Decr1 and Ivd provide potential molecular targets for deep exploration of immunometabolism in DCM. There are some limitations to our study. Firstly, we have only validated the hub genes in the DCM rats and lack the support of clinical data. Secondly, although a rigorous bioinformatics analysis was conducted in the present study, we did not conduct further experiments to verify the effects of mitochondrial metabolism genes on the immune microenvironment and cardiac function. Hence, the specific mechanism of immunometabolism regulation in DCM still needs to be further explored in vivo and in vitro. This novel direction will be the focus of our subsequent study.

## Conclusions

In summary, we identified the differences of mitochondrial related genes and immune cell infiltration between DCM and CON by comprehensive bioinformatics analysis. We found crosstalk between mitochondrial metabolism and immune infiltration in DCM for the first time. Four hub genes were screened and verified, among which Pdk4, Hmgcs2 and Decr1 were highly expressed in DCM, while Ivd was low. Most importantly, Pdk4, Hmgcs2 and Decr1 were positively correlated with Marginal Zone B and negatively correlated with DC cells; Ivd was positively correlated with CD8 Tem. These findings suggest that Pdk4, Hmgcs2, Decr1 and Ivd are co-regulatory molecules of immunometabolism in DCM. Besides, the infiltration differences of Marginal Zone B, Marginal Zone B and CD8Tem play an important role in the pathophysiology of DCM.

## Supplementary Information

**Additional file 1: Figure S1.** Box-plot of GSE4745, GSE5606 and GSE6880.

**Additional file 2: Table S1.** Data cohort characteristics.

**Additional file 3: Table S2.** Results of GSEA analysis.

**Additional file 4: Table S3.** GO enrichment analyses of DEGs from GSE4745, GSE5606 and GSE6880.

**Additional file 5: Table S4.** KEGG pathway enrichment analyses of DEGs from GSE4745, GSE5606 and GSE6880.

**Additional file 6: Table S5.** MitoDEGs in GSE4745, GSE5606 and GSE6880.

**Additional file 7: Table S6.** Hub MitoDEGs explored by MCODE and CytoHubba.

**Additional file 8: Table S7.** Hub MitoDEGs regulated by TF.

**Additional file 9: Table S8.** Hub MitoDEGs regulated by miRNAs.

**Additional file 10: Table S9.** Infiltration of immune cell types compared between the DCM and CON.

**Additional file 11: Table S10.** Relationship between hub MitoDEGs and immune cells.

**Additional file 12: Table S11.** Primers sequence of hub MitoDEGs.

## Data Availability

The datasets analysed during the current study are available in the GEO database (https://www.ncbi.nlm.nih.gov/geo/)and MitoCarta3.0 (http://www.broadinstitute.org/mitocarta), which is an updating, openly available for free download, authoritative database of mitochondrial proteome including sub-mitochondrial localization and MitoPathway annotations.

## Abbreviations

DCM: Diabetic cardiomyopathy
DEGs: Diferentially expressed genes
GSEA: Gene set enrichment analysis
GO: Gene ontology
KEGG: Kyoto Encyclopedia of Genes and Genomes
MitoDEGs: Mitochondria-related DEGs
PPI: Protein–protein interaction
HF: Heart failure
TF: Transcription factors
Pdk4: Pyruvate dehydrogenase kinase 4
Hmgcs2: 3-Hydroxy-3-methylglutaryl-CoA synthase 2
Decr1: 2,4-Dienoyl-CoA reductase 1
Ivd: Isovaleryl-CoA dehydrogenase
DM: Diabetes mellitus
LV: Left ventricular
CON: Control
STZ: Streptozotocin
LVEF: Left ventricular ejection fraction
FS: Fraction shortening
LVIDs: Left ventricular internal diameters at systole
LVIDd: Left ventricular internal diameters at diastole
BP: Biological process
CC: Cellular component
MF: Molecular function
HW: Heart weight
BW: Body weight
TL: Tibia length
MAM: Mitochondria-associated ER membranes
