# Supplementary figures and images for: Role of mitochondrial metabolic disorder and immune infiltration in diabetic cardiomyopathy: new insights from bioinformatics analysis
Source: J Transl Med. 2023 Feb 1;21:66. doi: 10.1186/s12967-023-03928-8 (PMC9893675; doi:10.1186/s12967-023-03928-8)

**a GSE4745, selected samples**

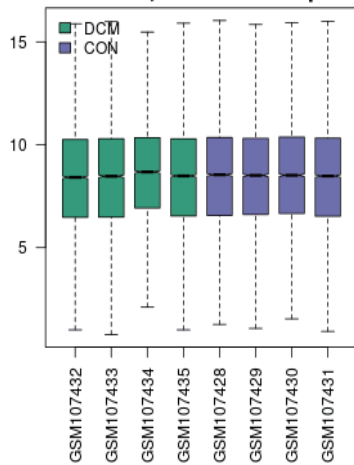

**b GSE5606**

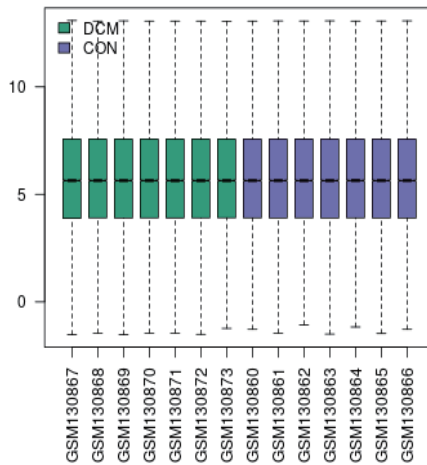

**c GSE6880**

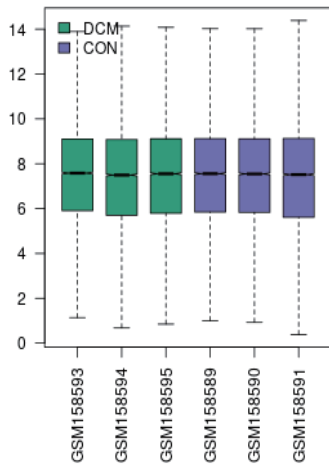

Supplement: Supplementary file 1 — Additional file 1: Figure S1. Box-plot of GSE4745, GSE5606 and GSE6880. [file 12967_2023_3928_MOESM1_ESM.pdf]
